# Elevated-Temperature Tribo-Corrosion Response of Eutectic High-Entropy Alloy

**DOI:** 10.3390/e28040391

**Published:** 2026-04-01

**Authors:** Jibril Shittu, Shristy Jha, Mayur Pole, Sundeep Mukherjee

**Affiliations:** 1 Department of Materials Science and Engineering, University of North Texas, Denton, TX 76203, USAsundeep.mukherjee@unt.edu (S.M.); 2 Lawrence Livermore National Laboratory, Livermore, CA 94550, USA; 3 Pacific Northwest National Laboratory, Richland, WA 99352, USA

**Keywords:** tribo-corrosion, elevated temperature, high-entropy alloy, reciprocating wear, corrosion

## Abstract

The combination of elevated temperature and tribo-corrosion leads to the accelerated degradation of structural components used in many extreme environments. Recently developed high-entropy alloys (HEAs) with multiple principal elements have the potential for superior degradation resistance compared with presently used structural alloys. Here, we demonstrate the microstructural stability, pitting resistance, and superior tribo-corrosion degradation resistance of the AlCoCrFeNi_2.1_ eutectic HEA in comparison with duplex stainless steel 2205 in deionized water (controlled low-ionic-strength electrolyte) at 25 °C, 50 °C and 100 °C. The AlCoCrFeNi_2.1_ HEA showed excellent microstructural stability and tribo-corrosion resistance at all three temperatures, an order-of-magnitude lower wear rate, and a lower coefficient of friction compared with duplex 2205 steel. The lowest wear volume loss and wear rate for both AlCoCrFeNi_2.1_ and duplex steel were recorded at 50 °C, which was attributed to temperature-assisted passivation and formation of a comparatively stable tribological surface condition. These results suggest superior performance of eutectic HEAs in tribo-corrosion applications compared with currently used dual-phase steels and motivate future evaluation in ion-containing industrial water chemistries.

## 1. Introduction

Complex concentrated alloys also known as high-entropy alloys (HEAs) represent a new paradigm in alloy development based on alloying multiple principal elements in (near) equimolar proportions [1,2]. This distinctive compositional strategy generates four fundamental phenomena: enhanced solid-solution strengthening, elevated configurational entropy, synergistic “cocktail effects” from element interactions, and severe lattice distortion due to atomic size differences [3,4]. Together, these mechanisms produce materials with exceptional combinations of mechanical strength, wear resistance, corrosion protection, oxidation resistance, and thermal stability [5,6]. The vast multi-component phase space gives ample opportunity for tuning degradation mechanisms by suitable compositional variations of HEAs for use in harsh conditions such as physiological environments [7,8] and advanced nuclear systems [9] to name a few.

The simultaneous action of wear and corrosion—termed tribo-corrosion—poses a critical challenge for mechanical systems operating in corrosive environments. With thermal activation, both degradation mechanisms are further accelerated. Elevated temperature is known to accelerate wear [10,11] and electrochemical reactions [12,13,14,15] in heat exchangers [16] and gas turbines [17]. Despite growing research interest separately on the wear and corrosion behavior of HEAs [18,19,20], there are very few reports and limited understanding of the combined tribo-corrosion response of alloys particularly at elevated temperatures. Multi-phase eutectic HEAs are of immense interest in this context because of their superior mechanical properties [21,22]. But there are no reports and limited scientific understanding on their tribo-corrosion response.

In this study, we conducted (i) static electrochemical tests to establish the baseline passivation behavior as a function of temperature, (ii) transient torch-based thermal shock to evaluate the susceptibility of the dual-phase microstructures to rapid heat-flux excursions and resulting phase redistribution, and (iii) elevated-temperature tribo-corrosion to quantify the coupled mechanical–electrochemical degradation under sliding. We report on the elevated-temperature tribo-corrosion response and microstructural stability of the AlCoCrFeNi_2.1_ eutectic HEA in comparison to commonly used duplex stainless steel (DS2205) in deionized water at room temperature (25 °C), 50 °C and 100 °C. These alloys were chosen because of their dual-phase microstructure, superior mechanical properties, corrosion resistance, and wear resistance [23,24]. While the wear, corrosion, mechanical properties, and microstructure of both alloys have been reported, there are no reports on their tribo-corrosion degradation, especially at elevated temperatures [25,26,27]. Together, these experiments connect microstructural integrity and passivation stability to tribo-corrosion performance.

## 2. Materials and Methods

### 2.1. Sample Preparation

The eutectic HEA with a nominal composition of AlCoCrFeNi2.1 was prepared from 99.99%-purity constituent elements using vacuum/argon arc melting. Duplex stainless steel DS2205 (UNS 31803), prepared according to ASTM A815 [28] specification by hot rolling of as-cast DS2205 purchased from James Duva Inc. (Columbia, NJ, USA). Cylinders of 6.35 mm diameter and 35 mm length were prepared from AlCoCrFeNi2.1 and DS2205 ingots using wire electrical discharge machining (EDM, Kent USA, Tustin, CA, USA) for the tribo-corrosion experiments. The flat bottom of each cylinder was polished with up to 1200-grit abrasive paper and then polished using 1 µm diamond suspension to obtain a mirror surface finish, followed by triple sonication in deionized water, methanol, and isopropyl alcohol. The cylinder sidewalls were sealed using acrylic to prevent galvanic coupling between the tribo-corrosion surface and cylinder walls during exposure to the electrolyte.

### 2.2. Microstructure Characterization and Surface Analysis

Microstructural characterization was performed using scanning electron microscopy (SEM, FEI Nova-NanoSEM 230™, FEI, New York, NY, USA). Samples cut along the same cross-sectional cylinder length were used to determine elemental composition, phase fraction, and crystal structure using energy-dispersive spectroscopy (EDS), electron backscatter diffraction (EBSD), and X-ray diffraction (XRD, Rigaku Ultima III, Rigaku, Tokyo, Japan), respectively. The surface topography of wear scars was examined using white-light interferometry. The wear volume loss was calculated from metrology analysis of 3D optical micrographs using Gwyddion software (version 2.70). Wear tracks were further analyzed in SEM to identify dominant material removal mechanisms.

### 2.3. Microstructural Integrity/Stability Under Rapid Thermal Cycling and High Heat Flux

The samples were sectioned to obtain smaller samples for corrosion testing, and the effect of the heat treatment on the phase morphology and evolution of both AlCoCrFeNi_2.1_ and DS2205 was determined using EBSD. To probe microstructural integrity under severe localized heating relevant to transient high-heat-flux excursions (thermal shock, steep thermal gradients, and rapid oxidation/erosion), an oxyacetylene torch exposure was performed on 6.35 mm diameter cylinders of both AlCoCrFeNi_2.1_ and DS2205. The torch tip was mounted 19 mm normal to the cylindrical height of both alloys. Both alloys were rapidly heated to approximately 1100 °C using the open flame and then rapidly quenched; this was repeated three times to compare microstructural change and phase redistribution under highly transient non-equilibrium conditions.

This torch-based exposure is not intended to represent equilibrium aging, equilibrium phase stability, or continuous-service capability at 1100 °C. Instead, it provides a comparative assessment of microstructural integrity and damage evolution under rapid thermal cycling and high heat flux. DS2205 is not designed for continuous service at 1100 °C, and its inclusion here serves as a baseline comparison against a widely used duplex structural alloy under the same transient thermal shock conditioning.

Following exposure, the cylinder samples were sectioned to obtain smaller samples for corrosion testing. The effect of thermal shock conditioning on phase morphology and evolution was characterized by using EBSD on the substrate (bulk) microstructure. Depth-resolved oxide scale thickness mapping, compositional profiling, or cross-sectional oxidation microstructure gradients were not pursued in this study because the objective is comparative tribo-corrosion performance after a consistent pre-conditioning step rather than oxidation kinetics characterization.

### 2.4. Elevated-Temperature Tribo-Corrosion Test

**Figure 1:**
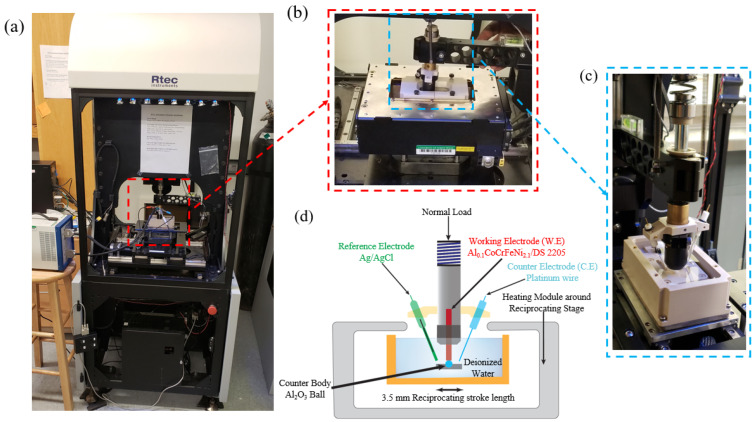
Tribo-corrosion experimental setup. The electrochemical cell was set-up on a linear reciprocating tribometer within a heating module to simulate elevated-temperature tribo-corrosion: (**a**) tribo-corrosion experimental setup with RTEC Universal tribometer coupled to a Gamry potentiostat; (**b**) tribo-corrosion and heating module assembly; (**c**) immersed tribo-corrosion cell and (**d**) schematic representation of the elevated-temperature tribo-corrosion setup.

Elevated-temperature tribo-corrosion experiments were performed using a linear reciprocating inverted ball-on-flat setup as shown in Figure 1. The reciprocating tribo-corrosion module was mounted on an RTEC Instruments tribometer (RTEC, San Jose, CA, USA) coupled with a Gamry potentiostat (Gamry, Warminster, PA, USA) (Figure 1a). Figure 1b shows the tribo-corrosion module mounted within the heating module, while Figure 1c shows the immersed tribo-corrosion cell. A 6.35 mm diameter alumina ball was held in a resin cast at the bottom with the top of the ball aligned with the center of the polished alloy cylinder. The sample and counter-body were held for 3600 s in deionized water to achieve open circuit potential (OCP) stability prior to sliding. The red terminal in Figure 1d indicates the working electrode (cylindrical AlCoCrFeNi2.1 or DS2205 sample); the blue terminal indicates the counter electrode (platinum wire); and the green terminal indicates the reference electrode (Ag/AgCl electrode). All three electrodes were placed within 10 mm of each other according to ASTM guidance for minimizing solution resistance artifacts in electrochemical tests (STP 1056) [29]. Briefly, 100 mL of deionized water was used to immerse samples, counter-body, and electrodes for each tribo-corrosion experiment. All tribo-corrosion tests were conducted at 25 °C, 50 °C, and 100 °C. The effect of normal load on tribo-corrosion performance was measured at each temperature using a 5 Hz frequency and 3.5 mm stroke length while varying the normal load to 5 N, 10 N, and 15 N for 1800 s of sliding after the 3600 s OCP stabilization period. The effect of the reciprocation frequency on tribo-corrosion performance was studied at each temperature by varying the frequency from 3 Hz to 5 Hz and 9 Hz while keeping the load fixed at 5 N. After the sliding test, the sample remained immersed in the electrolyte to measure the OCP for an additional 300 s to assess surface repassivation after sliding cessation. Three tribo-corrosion tests were performed for each condition to obtain the statistical average and standard deviation of wear volume loss and wear rate. DI water was intentionally selected as a controlled, low-ionic-strength electrolyte to minimize solution-chemistry variability and isolate the effect of temperature on depassivation and repassivation during sliding rather than conflating results with chloride-driven pitting or conductivity-dependent kinetics. Performance in chloride-/sulfate-containing waters is a logical next step for application-specific evaluation. Tests at 100 °C were conducted in a covered electrochemical cell equipped with a reflux condenser so that evaporated water condensed and returned to the cell. This prevented net evaporation and maintained a stable liquid level and immersion condition throughout the test. Electrolyte temperature was regulated using a feedback-controlled heating setup, with temperature monitored in the electrolyte near the contact zone. The reflux configuration minimizes bubble-related disturbance and liquid-level fluctuations that can occur near the boiling point.

## 3. Results and Discussion

### 3.1. Microstructure and Electrochemical Characterization

**Figure 2:**
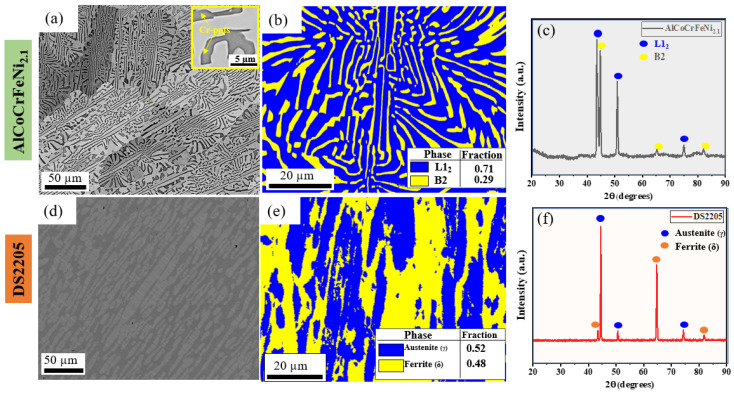
Microstructural characterization of AlCoCrFeNi_2.1_ HEA and DS2205. (**a**) BSE image of AlCoCrFeNi_2.1_ HEA showing L1_2_ and B2 phases in light and dark contrasts, respectively; (**b**) EBSD phase map showing AlCoCrFeNi_2.1_ HEA volume fraction of the two phases; (**c**) X-ray diffraction pattern showing a dual-phase AlCoCrFeNi_2.1_ HEA crystal structure; (**d**) BSE image of DS2205 showing austenite and ferrite phases in light and dark contrasts, respectively; (**e**) EBSD phase map showing volume fraction of the two phases; (**f**) X-ray diffraction pattern showing a dual-phase DS2205 crystal structure.

Microstructural characterizations of both AlCoCrFeNi_2.1_ HEA and DS2205 are shown in Figure 2. Both AlCoCrFeNi_2.1_ HEA and DS2205 ferritic–austenitic stainless steel were chosen because of their similarity of having both BCC (B2) and FCC (L1_2_) phases. Although the AlCoCrFeNi_2.1_ HEA is made up of the ordered derivative phases, its underlying lattice geometry remains similar. The backscattered secondary electron (BSE) micrograph shown in Figure 2a represents the as-cast microstructure of the AlCoCrFeNi_2.1_ HEA. The microstructure consists of a lamellar arrangement of dark and light contrast solid solution phases that correspond to B2 and L1_2_ phases, respectively [27]. The B2 phase contained nano-sized precipitates (white spots) as shown in the high-magnification image (inset in Figure 2a). These nano-precipitates were previously reported to be Cr-rich [30,31]. This fine distribution of nano-sized precipitate throughout the B2 lamellae ensures that chromium is uniformly available across the microstructure rather than concentrated in coarse phases. This homogeneous Cr distribution promotes more-consistent passive film coverage and formation during tribology. Figure 2b shows the EBSD phase map with lamellar structure of B2 (yellow color) and L1_2_ (blue color) encompassing the B2 phase. The volume fraction was determined to be approximately 71% for the L1_2_ phase and 29% for the B2 phase. The volume fraction and microstructure in this study are consistent with prior AlCoCrFeNi_2.1_ HEA studies [32]. Reported phase fractions in AlCoCrFeNi_2.1_ can vary with processing history (as-cast vs. homogenized/aged), segregation state, and the quantification method (e.g., EBSD thresholding vs. bulk-averaged XRD) [33,34]. The phase fractions reported here are specific to the as-cast condition examined and were obtained using consistent EBSD acquisition and cleanup parameters across all samples. The B2 lamellae with thicknesses of ~2–3 µm are distributed in the L1_2_ matrix. Figure 2c shows the X-ray diffraction (XRD) of the AlCoCrFeNi_2.1_ HEA, with the higher-intensity peaks indicating the L1_2_ phase and lower-intensity peaks indicating the B2 phase (consistent with EDS phase fraction analysis). The backscattered secondary electron (BSE) micrograph of DS2205 ferritic–austenitic stainless steel in Figure 2d shows elongated ferritic and austenitic phases. The darker contrast phase is the δ-phase (Ferrite-BCC), and the lighter contrast is the γ-phase (Austenite-FCC). Quantification from the EBSD phase map in Figure 2e shows that the δ-phase had 48% and the γ-phase had 52% (approximately an equal amount) of the lamellar structures. XRD analysis of DS2205 in Figure 2f shows the peak intensity of both the δ-phase and the γ-phase to agree with the microstructure and EBSD phase map.

**Figure 3:**
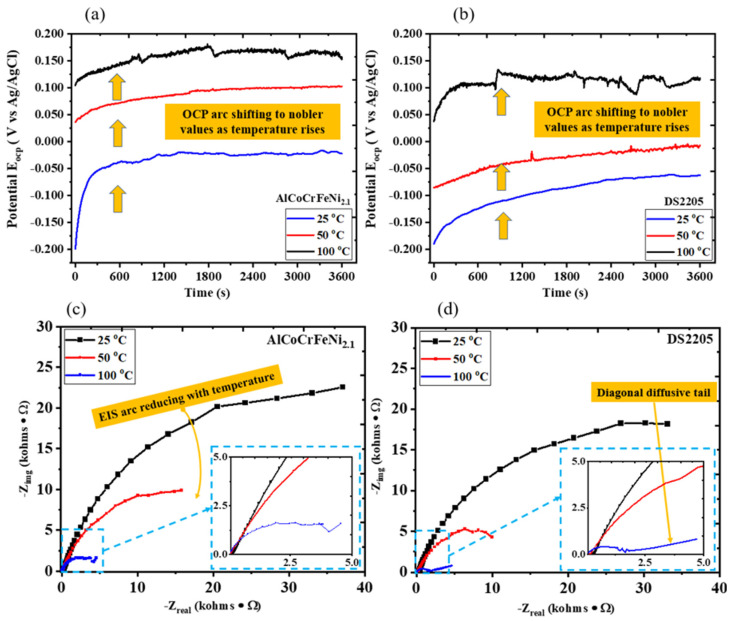
Electrochemical characterization of AlCoCrFeNi_2.1_ HEA and DS2205. (**a**) OCP plot for AlCoCrFeNi_2.1_ HEA at room temperature, 50 °C and 100 °C for 3600 s; (**b**) OCP plot for DS2205 at room temperature, 50 °C and 100 °C for 3600 s; (**c**) Nyquist plot for AlCoCrFeNi_2.1_ HEA at room temperature, 50 °C and 100 °C (inset showing zoomed-in section of the lower frequency); (**d**) Nyquist plot for DS2205 at room temperature, 50 °C and 100 °C (inset showing zoomed-in section of the lower frequency). The Nyquist arc diameter for both alloys decreases as temperature increases, indicating a reduction in overall interfacial impedance and charge transfer resistance at elevated temperature. DS2205 exhibits a low frequency diagonal diffusive tail consistent with a diffusion-controlled contribution to the overall impedance.

The electrochemical characterizations of the AlCoCrFeNi_2.1_ HEA and DS2205 are summarized in Figure 3a–d. EIS was used as a baseline comparison of passive behavior under static immersion conditions prior to sliding. Equivalent-circuit fitting is not presented because passive-alloy spectra at elevated temperatures can exhibit overlapping time constants, and non-ideal capacitive behavior can lead to non-unique fitted parameters without independent validation. EIS is used here primarily to establish qualitative consistency of the temperature-dependent passivation behavior rather than to develop a unique mechanistic circuit model. The OCP of the AlCoCrFeNi_2.1_ HEA at room temperature (25 °C), 50 °C and 100 °C shown in Figure 3a indicates a consistent shift toward nobler values as temperature increases. This may be attributed to temperature-assisted passivation as the OCP at 50 °C stabilized faster than at room temperature. Although the OCP shifts to nobler values as the temperature increases from 50 °C to 100 °C, the OCP curve at 100 °C shows greater instability than the OCP at 50 °C and 25 °C. Occasional spikes and dips in the OCP at 100 °C may be attributed to the rupture of the surface passive layer before gradual repassivation. Figure 3b shows the OCP of DS2205 at 25 °C, 50 °C and 100 °C with similar trends to those for the AlCoCrFeNi_2.1_ HEA. But the OCP for DS2205 showed a greater degree of instability, and the steady-state values were significantly less noble than those for AlCoCrFeNi_2.1_ HEA. Electrochemical impedance spectroscopy (EIS) for the AlCoCrFeNi_2.1_ HEA shown in the Nyquist plot in Figure 3c indicates a consistent reduction in arc radius as temperature increases, indicating a reduction in polarization resistance with increase in temperature. The Nyquist plot for DS2205 in Figure 3d follows a similar trend with a decrease in arc radius as temperature increases. A comparison of the arc radius at the same temperature for both AlCoCrFeNi_2.1_ HEA and DS2205 indicates that the AlCoCrFeNi_2.1_ HEA has an overall higher polarization resistance than DS2205 at all temperatures. Notably, the Nyquist plot for DS2205 at 100 °C was characterized by a semicircular plot with a diagonal diffusive tail at a lower frequency. The semicircular region on the left signifies the coupling between double-layer capacitance and surface kinetic effects at frequencies faster than the physical process of diffusion. The diagonal diffusive tail (shown in the Figure 3d inset) indicates diffusion effects at higher temperatures and lower frequencies. This diagonal diffusive tail supports the OCP behavior seen for DS2205 at a higher temperature. Overall, the AlCoCrFeNi_2.1_ HEA exhibited more-stable surface passivation than DS2205 at all temperatures investigated.

### 3.2. Microstructural Integrity After Rapid Thermal Shock Conditioning

**Figure 4:**
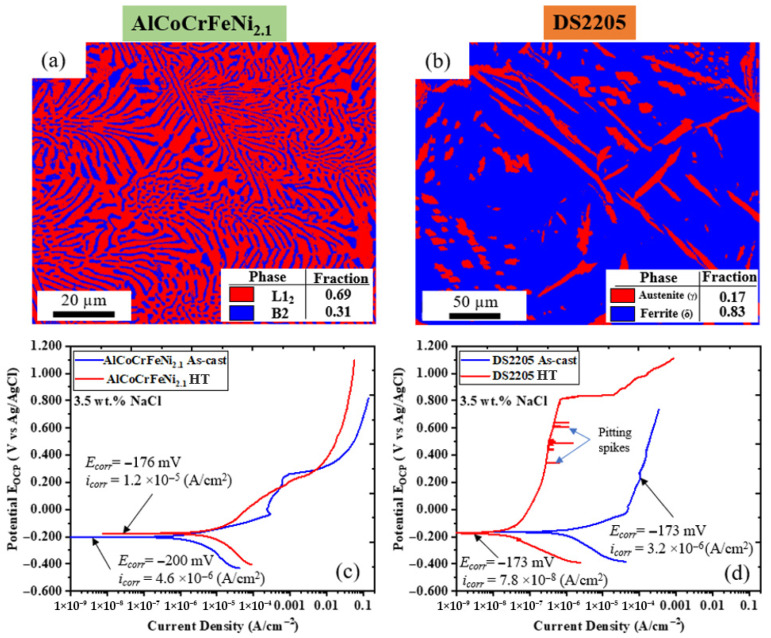
Heat-treated AlCoCrFeNi_2.1_ HEA and DS2205 microstructure and corrosion resistance: (**a**) EBSD phase map showing AlCoCrFeNi_2.1_ HEA volume fraction of the L1_2_ and B2 phases; (**b**) EBSD phase map showing DS2205 volume fraction of the δ-phase (Ferrite-BCC) and γ-phase (Austenite-FCC); (**c**) potentiodynamic polarization curve of as-cast and heat-treated AlCoCrFeNi_2.1_ HEA; (**d**) potentiodynamic polarization curve of as-cast and heat-treated DS2205.

The EBSD phase map and potentiodynamic polarization plots for AlCoCrFeNi_2.1_ HEA and DS2205 rapid thermal shock conditioning are shown in Figure 4. EBSD phase characterization of the thermally shocked AlCoCrFeNi_2.1_ HEA identified phase fractions of 69% L1_2_ and 31% B2 (Figure 4a). This indicates little to no significant microstructural transformation in phase fraction after thermal shock conditioning (as-cast was 71% L1_2_ and 29% B2 phase fractions). In contrast, DS2205 showed 17% γ-phase (Austenite-FCC) and 83% δ-phase (Ferrite-BCC) after thermal shock conditioning as seen in Figure 4b. This indicates a significant difference compared with the as-cast microstructure of 48% δ-phase and 52% γ-phase. Therefore, the AlCoCrFeNi_2.1_ HEA exhibits substantially higher microstructural integrity under rapid thermal shock than DS2205. This result is consistent with the microstructural stability predicted for high-entropy alloys from the four core effects. Evaluations of corrosion resistance by potentiodynamic polarization tests of as-cast and thermally shocked samples of AlCoCrFeNi_2.1_ HEA and DS2205 are shown in Figure 4c,d. Potentiodynamic polarization of AlCoCrFeNi_2.1_ HEA in 3.5 wt.% NaCl in Figure 4c shows a slight shift towards a nobler potential from an *E_corr_* (corrosion potential) of −200 mV to −176 mV and a modest shift in *i_corr_* (corrosion current density) from 4.6 × 10^−6^ (A/cm^2^) to 1.2 × 10^−5^ (A/cm^2^) for as-cast and heat-treated AlCoCrFeNi_2.1_ HEA, respectively. This slight shift (−24 mV) towards a nobler potential is indicative of a thermodynamically more stable surface. Corrosion studies by Dong et al. [35] showed that corrosion occurs preferentially in the BCC phase of AlCoCrFeNi_2.1_ HEA; therefore, an increase in BCC of 2% can be attributed to a slight increase in corrosion potential. For the potentiodynamic polarization of DS2205 in Figure 4d, the *E_corr_* was relatively unchanged at ~−173 mV for both for as-cast and heat-treated DS2205. A major shift towards lower *i_corr_* (corrosion current density) was seen after heat treatment from 3.2 × 10^−6^ (A/cm^2^) for as-cast to 7.8 × 10^−8^ (A/cm^2^) for rapid thermal-shock-conditioned DS2205. These two order-of-magnitude shifts may be attributed to microstructural transformation resulting in the increase in the δ-phase (Ferrite-BCC), which is known to have a better corrosion resistance than the γ-phase (Austenite-FCC). This microstructural transformation resulting in an island microstructure of the γ-phase in a δ-phase matrix is indicative of the unstable austenitic–ferritic microstructure. Although, corrosion current density may show better corrosion resistance for the heat-treated sample, the susceptibility to localized corrosion in the form of pitting can be seen on the potentiodynamic polarization curve. Pitting corrosion, seen as corrosion current density spikes on the red curve, is indicative of passive film degradation and galvanic corrosion resulting from the Cr-rich and Cr-depleted regions in the δ-phase matrix previously reported [23]. Overall, the AlCoCrFeNi_2.1_ HEA showed a more stable microstructure and potentiodynamic polarization curve than DS2205.

### 3.3. Effect of Temperature on Tribo-Corrosion

**Figure 5:**
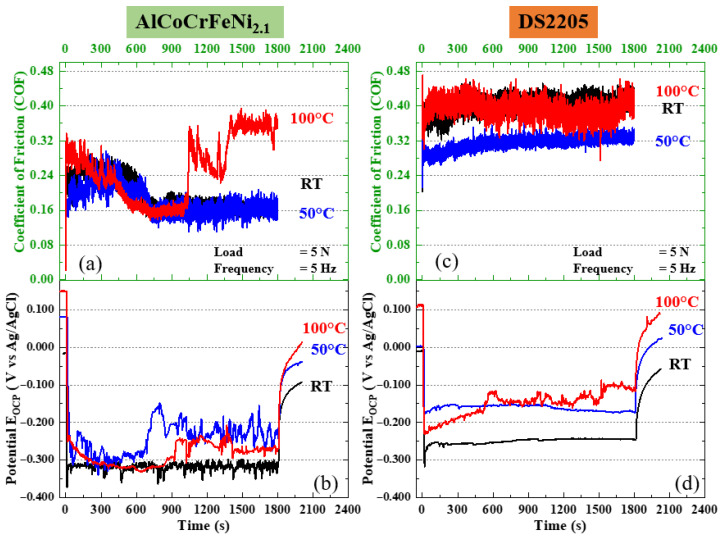
(**a**) Coefficient of friction and (**b**) open circuit potential (*E*_ocp_) as a function of temperature at 5 Hz frequency and 5 N load for AlCoCrFeNi_2.1_ HEA; (**c**) COF and (**d**) OCP as a function of temperature at 5 Hz frequency and 5 N load for DS2205.

To investigate the effect of temperature on the tribo-corrosion response of AlCoCrFeNi_2.1_ and DS2205, the OCP and COF were recorded simultaneously during reciprocating sliding at a load of 5 N and frequency of 5 Hz in DI water at 25 °C, 50 °C, and 100 °C, as shown in Figure 5. These in-test electrochemical and tribological signatures are interpreted together with post-test wear morphologies (Figure 7) to clarify how temperature influences the dominant material removal mechanisms through changes in depassivation, repassivation, and tribo-layer stability. For AlCoCrFeNi_2.1_, the COF in Figure 5a at 25 °C and 50 °C shows similar magnitudes with average values of 0.195 ± 0.012 and 0.181 ± 0.015, respectively. The relatively low and stable COF at 25 °C corresponds to the predominantly two-body abrasive morphology in Figure 7a, characterized by fine grooves aligned along the sliding direction with minimal pile-up. Under this condition, sliding repeatedly disrupts the passive film locally, but the wear track morphology suggests that debris generation and entrainment are limited, and material removal is dominated by micro-ploughing/micro-cutting rather than by severe adhesive transfer or extensive third-body abrasion. At 50 °C, AlCoCrFeNi_2.1_ exhibits its lowest COF and the most stable frictional response after the run-in period. This friction behavior is consistent with the polished wear track observed in Figure 7b, which shows reduced grooving and a comparatively smooth contact region. The polishing morphology at 50 °C indicates a more stable near-surface condition during sliding, which is consistent with temperature-assisted passivation promoting rapid repair of sliding-induced depassivation sites. In this regime, the formation and retention of a thin passive film and/or a mechanically compacted tribo-layer can reduce shear strength at the interface and suppress aggressive abrasion, yielding the observed reduction in COF and the minimized wear response at 50 °C. At 100 °C, AlCoCrFeNi_2.1_ initially shows a COF trend similar to those at 25 °C and 50 °C but transitions after approximately 1000 s to a higher and more-variable friction level with an average COF of 0.254 ± 0.094. This transition in COF aligns with the wear morphology at 100 °C (Figure 7c), where the wear track contains discontinuous tribo-layer patches and regions of dislodged oxide/tribo-layer material in the central contact area, while the track edges appear comparatively polished. This morphology suggests oxidative wear and tribo-layer instability at the highest temperature. Although elevated temperature accelerates repassivation kinetics, it can also promote thicker, more-brittle, or less-adherent oxide/tribo-layers that are repeatedly formed and removed during reciprocating sliding. The cyclic formation, fracture, and ejection of these patches can increase friction variability and contribute to localized three-body interactions even if the dominant appearance remains patchy oxidative wear rather than deep ploughing.

The OCP response of AlCoCrFeNi_2.1_ during tribo-corrosion (Figure 5b) further supports these temperature-dependent wear regimes. At 25 °C, the OCP is comparatively steady during sliding with minor transients, consistent with a near-steady balance between depassivation and slower repassivation and consistent with the relatively uniform abrasive grooving in Figure 7a. At 50 °C and 100 °C, the OCP shows more-pronounced undulations and shifts toward nobler values after the run-in period. This behavior indicates faster repassivation kinetics and a dynamically evolving surface film during sliding. In particular, the stronger OCP instability at 100 °C is consistent with repeated rupture and repair events associated with the discontinuous tribo-layer patches seen in Figure 7c. After sliding stops, the repassivation segment follows the expected temperature dependence (faster recovery at higher temperature), consistent with thermally activated surface film repair.

For DS2205, the COF during tribo-corrosion in Figure 5c is substantially higher than for AlCoCrFeNi_2.1_ at all temperatures, averaging 0.403 ± 0.018 at 25 °C, 0.315 ± 0.014 at 50 °C, and 0.394 ± 0.021 at 100 °C. The high COF at 25 °C is consistent with the wider wear track and deeper abrasive grooves observed in Figure 7d, indicating more-severe two-body abrasion and greater material displacement. The larger track width and groove depth suggest higher susceptibility to micro-ploughing and micro-cutting, which increases both friction and wear relative to the HEA under the same conditions. At 50 °C, DS2205 exhibits its lowest COF, which corresponds to the comparatively smooth, polishing-type wear track in Figure 7e. Although displaying similar smooth polishing-type wear, the DS2205 wear track remains wider than the HEA track. The reduced grooving and smoother contact are consistent with improved passivation stability at 50 °C producing an interface that is less prone to severe abrasion. The visible duplex microstructure within the wear track and the pronounced pile-up at the edges are consistent with ductile deformation and material flow accompanying polishing wear. This is consistent with an “optimum passivation window” at 50 °C in DI water, where film repair is sufficiently rapid to suppress severe abrasion while the film remains stable enough to avoid extensive brittle debris generation. At 100 °C, the DS2205 COF increases again toward values closer to 25 °C. The wear morphology in Figure 7f indicates a transition toward three-body abrasion, with ploughing features consistent with entrapped debris and re-circulated third-body particles. The noticeably increased edge pile-up at 100 °C relative to 50 °C suggests thermal softening and enhanced plastic flow in DS2205, which can increase debris production and facilitate third-body entrapment. In addition, the OCP response for DS2205 (Figure 5d) trends toward a nobler potential at 100 °C during sliding, indicating active film formation, but the simultaneous development of three-body abrasion implies that any oxide/corrosion product film formed is not mechanically stable under sliding. Instead, film formation and breakdown can generate abrasive particles that promote ploughing and sustain higher friction.

Overall, combining the in-test OCP and COF measurements (Figure 5) with post-test wear morphologies (Figure 7) indicates that temperature alters tribo-corrosion response primarily by shifting the balance between (i) abrasive wear under less-effective or slower film repair (dominant at 25 °C), (ii) a polishing regime associated with temperature-assisted passivation and a stable near-surface condition (dominant at 50 °C), and (iii) film/tribo-layer instability and oxidative or debris-assisted wear at the highest temperature (100 °C). Across all temperatures, AlCoCrFeNi_2.1_ maintains a lower COF and more-stable wear morphology than DS2205, consistent with its more-robust passivation behavior and higher resistance to severe abrasion and debris-driven degradation under elevated-temperature tribo-corrosion conditions.

### 3.4. Dynamic Load and Frequency Tribo-Corrosion Degradation Rate

**Figure 6:**
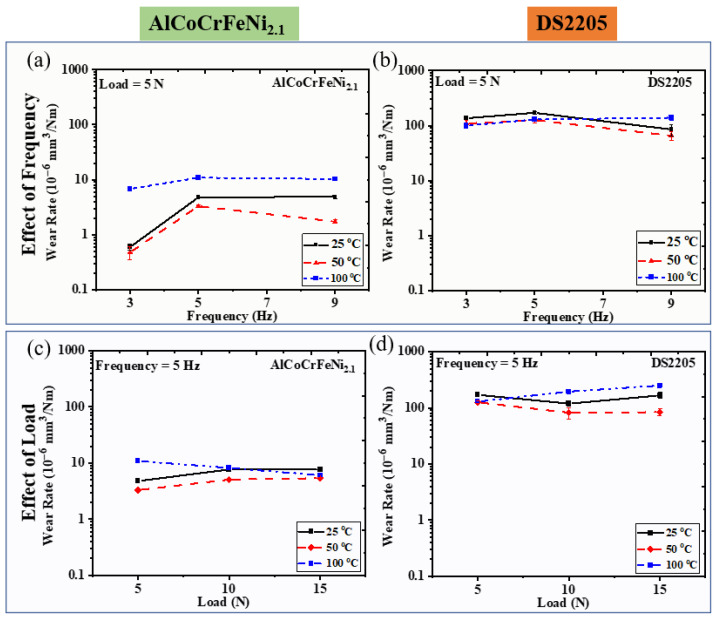
Wear rate at RT, 50 °C and 100 °C as a function of reciprocating frequency and load for of AlCoCrFeNi_2.1_ HEA and DS2205; (**a**) wear rate for of AlCoCrFeNi_2.1_ HEA at 3 Hz, 5 Hz and 9 Hz; (**b**) wear rate for of DS2205 at 3 Hz, 5 Hz and 9 Hz; (**c**) wear rate for of AlCoCrFeNi_2.1_ HEA at 5 N, 10 N and 15 N; (**d**) wear rate for of DS2205 at 5 N, 10 N and 15 N.

To simulate dynamic degradation conditions at elevated temperature, varying loads from 5 N to 10 N and 15 N and varying reciprocating frequencies of 3 Hz, 5 Hz and 9 Hz were applied to the tribo-corrosion test. The resultant wear rates for the effect of frequency and load for both AlCoCrFeNi_2.1_ HEA and DS2205 are shown in Figure 6 with a strong dependence on temperature for the wear rate experienced by both alloys. For the effect of frequency, Figure 6a shows the wear rate of AlCoCrFeNi_2.1_ HEA at reciprocating frequencies of 3 Hz, 5 Hz, and 9 Hz at 25 °C, 50 °C and 100 °C. It can be observed that increasing the frequency from 3 Hz to 5 Hz led to a steeper increase in slope for wear rate at all three temperatures but further increase from 5 Hz to 9 Hz led to a reduced slope in wear rate. This trend may be attributed to the low reciprocating frequency of 3 Hz allowing for ample time for repassivation of the worn surface between cycles. But the higher reciprocation frequency of 5 Hz allowed insufficient time between reciprocating cycles, leading to less repassivation and higher wear rates. The decrease in the slope from 5 Hz to 9 Hz may be attributed to reduced material removal as the surface interaction time decreased with increasing reciprocating frequency. The wear rate from 5 Hz to 9 Hz follows a decreased-interaction and decreased-material-removal model observed at higher oscillating frequency for reciprocating wear. For the DS2205 wear rate in Figure 6b, wear rate reduced with increasing temperature as the material removal mechanism changed. For the effect of normal load on the wear rate of AlCoCrFeNi_2.1_ HEA in Figure 6c, increasing the load from 5 N to 15 N had a similar trend at both 25 °C and 50 °C. But a reverse trend was observed with the wear rate as the load was increased at 100 °C, this can be attributed to the unstable passive layer at a higher temperature and possible near-surface softening as increasing load leads to increased frictional heating of the contact surface. For the effect of load on DS2205 in Figure 6d, similar behavior was observed at both 25 °C and 50 °C with a decrease in wear rate as load increased from 5 N to 10 N and a further increase in wear rate going from 10 N to 15 N. This change in wear rate can be attributed to the changing contact mechanics of the ductile DS2205 as the load moves from 5 N to 15 N. At 100 °C the wear rate increased drastically from 5 N to 15 N, possibly due to the unstable passive layer and thermal softening at 100 °C. Overall, the AlCoCrFeNi_2.1_ HEA exhibited approximately one order-of-magnitude lower and more-stable tribo-corrosion wear rates than DS2205 across all temperatures, frequencies and loads investigated.

### 3.5. Wear Mechanism of AlCoCrFeNi_2.1_ HEA and DS2205

**Figure 7:**
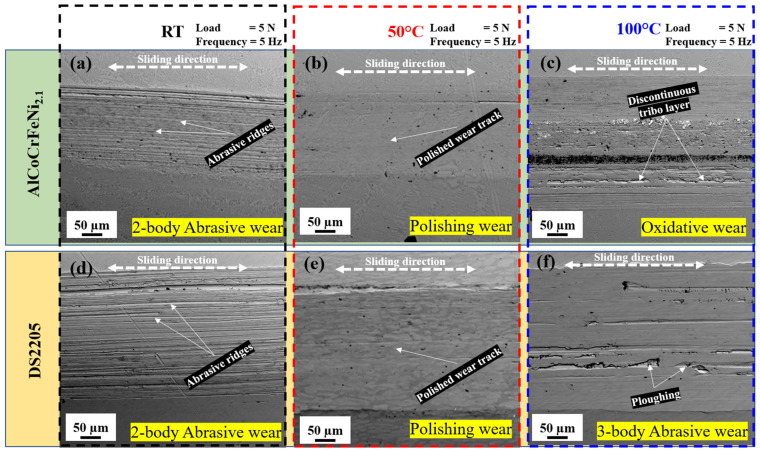
Scanning electron micrograph showing the material removal mechanism for AlCoCrFeNi_2.1_ HEA and DS2205 tribo-corrosion at RT, 50 °C and 100 °C; (**a**) AlCoCrFeNi_2.1_ HEA wear track at RT; (**b**) AlCoCrFeNi_2.1_ HEA wear track at 50 °C; (**c**) AlCoCrFeNi_2.1_ HEA wear track at 100 °C; (**d**) DS2205 wear track at RT; (**e**) DS2205 wear track at 50 °C; (**f**) DS2205 wear track at 100 °C.

The surface morphologies of AlCoCrFeNi_2.1_ HEA and DS2205 wear tracks after tribo-corrosion at 25 °C, 50 °C and 100 °C with 5 Hz frequency and 5 N normal load are shown in Figure 7. As the temperature changed from 25 °C to 50 °C and 100 °C, the wear mechanism significantly changed with passivation layer stability at the temperatures investigated. Figure 7a shows the surface morphology of AlCoCrFeNi_2.1_ HEA at 25 °C with a characteristic two-body abrasive wear deduced from the multiple abrasive grooves aligned along the reciprocating sliding direction. The abrasive ridges appear to be shallow without pile-up on the edge of the wear track. The two-body abrasive wear track at 25 °C supports the results previously seen for the COF and wear rate. In Figure 7b, the surface morphology of AlCoCrFeNi_2.1_ HEA at 50 °C shows characteristic polishing wear without significant grooves along the sliding direction. This polished wear track is consistent with the low wear rate, low wear volume loss and reduced COF observed at 50 °C for AlCoCrFeNi_2.1_ HEA. Figure 7c, the surface morphology of AlCoCrFeNi_2.1_ HEA at 100 °C shows a discontinuous tribo-layer with multiple patches of dislodged oxides in the middle of the contact area and a polished surface towards the edge of the wear track. This morphology at 100 °C indicates oxidative wear at higher temperatures, and the unstable COF, which increases as tribo-corrosion proceeds, supports the oxidative wear characteristics. For DS2205 surface morphology at 25 °C shown in Figure 7d, two-body abrasive wear was observed with similar morphology to that in AlCoCrFeNi_2.1_ HEA. The wear track for DS2205 was wider and had a deeper groove than the AlCoCrFeNi_2.1_ HEA wear track. The characteristic wider track and deeper grooves account for the order-of-magnitude increase in wear rate and the increased COF reported in the previous sections. At 50 °C, the tribo-corrosion wear morphology shown in Figure 7e can be characterized as polishing wear. The wear track appears to be polished while maintaining a visible microstructure of γ-phase and δ-phase within the wear track. The considerable pile-up on the edge of the wear track is consistent with the ductility of DS2205, and the significantly low COF at 50 °C is consistent with the smooth and polished wear track. The wear track is significantly wider than the AlCoCrFeNi_2.1_ HEA wear track at 50 °C, which accounts for the increased wear rate of DS2205 at 50 °C when compared with AlCoCrFeNi_2.1_ HEA. At 100 °C in Figure 7f, the surface morphology of DS2205 revealed a three-body abrasive wear mechanism of material removal. The ploughing seen on the wear track is characteristic of a trapped third body dislodged during the tribo-corrosion event. This leads to an incomplete grove as can be seen on the wear track. The edge pile-up at 100 °C is noticeably more than the edge pile-up at 50 °C, this suggests thermal softening at higher temperatures for DS2205 elevated-temperature tribo-corrosion. This wear morphology is consistent with the increased COF at 100 °C for DS2205, also consistent with a reduced wear rate just slightly higher than the wear rate at 50 °C. The width of the wear track is significantly larger than the wear track of AlCoCrFeNi_2.1_ HEA at 100 °C, and the micro-cutting and ploughing of DS2205 removes more material than the dislodged tribo-layer volume. The simultaneously recorded OCP in Section 3.3 provides an electrochemical signature of the evolving surface state during sliding. Conditions showing a comparatively stable OCP during the wear interval indicate a near-steady balance between depassivation and repassivation within the wear track, consistent with predominantly abrasive or polishing wear without large intermittent film breakdown events. In contrast, pronounced OCP undulations and spikes reflect repeated passive-film rupture and re-formation, which aligns with the patchy tribo-layer and dislodged oxide features observed at 100 °C. The polishing-dominated tracks at 50 °C for both alloys coincide with the minimum wear rates and comparatively stable electrochemical response, supporting a temperature-assisted passivation regime that reduces third-body abrasion and suppresses severe ploughing. Overall, AlCoCrFeNi_2.1_ HEA showed a much more stable wear morphology, smaller worn area, and ultimately better tribo-corrosion resistance than DS2205 at all the temperatures and conditions investigated.

## 4. Conclusions

In summary, the microstructural stability and elevated-temperature tribo-corrosion of a eutectic high-entropy alloy (AlCoCrFeNi_2.1_) were studied in deionized water to compare with those of DS2205 under identical conditions. AlCoCrFeNi_2.1_ HEA showed excellent microstructural stability and corrosion resistance with only 2% microstructural transformation in the L1_2_ phase to B2 phase, while DS2205 showed ~35% microstructural transformation from the γ-phase (austenite) into the δ-phase (ferrite) under the same conditioning. During tribo-corrosion testing at 25 °C, 50 °C and 100 °C, AlCoCrFeNi_2.1_ HEA exhibited stable depassivation–repassivation behavior, lower COF, and an approximately one order-of-magnitude lower wear rate than DS2205 across all temperatures, loads, and frequencies investigated. The trend in wear volume loss during tribo-corrosion was 50 °C < 25 °C < 100 °C for both alloys, consistent with an optimum passivation and tribological stability window near 50 °C under the present DI water conditions. The observed temperature dependence reflects the coupled influence of mechanical wear, corrosion, and their synergy under sliding. Temperature affects repassivation kinetics and the stability of tribo-layers and corrosion products, thereby shifting the wear mode from abrasive toward polishing behavior near 50 °C and toward less-stable oxide/tribo-layer disruption at 100 °C. Overall, AlCoCrFeNi_2.1_ HEA showed a more stable wear morphology and a smaller worn area, resulting in substantially improved tribo-corrosion resistance compared with DS2205.

## Data Availability

The original contributions presented in this study are included in the article. Further inquiries can be directed to the corresponding author.
